# Editorial: Neonatal outcomes - what about sex, race(ism) and social determinants of health?

**DOI:** 10.3389/fped.2025.1723226

**Published:** 2025-11-13

**Authors:** Rachana Singh

**Affiliations:** Department of Pediatrics, Tufts University School of Medicine, Boston, MA, United States

**Keywords:** sex, race, SDOH, neonates, genetics

While the majority of newborns are born healthy at term gestational age, advancements in medical/surgical interventions in the field of neonatal care have allowed many newborns with complex disorders at birth to survive, albeit with the potential for long-term morbidities and at risk for neurodevelopmental deficits (1). This is evident in the increased survival of preterm infants with complications associated with preterm birth, along with term infants with congenital medical and surgical disorders (2–4). Some growing evidence suggests that variations in neonatal outcomes cannot be explained solely by illness severity and clinical interventions. There are likely additional factors at play that can negatively impact outcomes, and identifying some modifiable risk factors could help mitigate the inequities in outcomes (5–7). Also, while it is well known that race is a social construct, it continues to be reported in ongoing studies leading to biases in care provision with resultant inequities in neonatal outcomes (8).

Currently, there is a limited body of literature highlighting the influence of neonatal sex, race(ism), and maternal Social Determinants of Health (SDoH) on neonatal outcomes. It is a common adage in Pediatrics that male infants will have relatively poorer outcomes compared to female infants, but the reason why is still unanswered. Historically, race has been tied to health outcomes, sending an erroneous message with a significant impact on the care provided to people of color and minorities. Finally, disparities in accessing healthcare based on one's socioeconomic status and the neighborhood one lives in are documented for adults and children. The aim of this research topic was to describe the scientific rationale for the role infant sex plays; how we can eliminate the influence of race(ism) on neonatal outcomes, and how we can effectively address SDoH-related modifiable factors through early identification and public health policies that support families and newborns affected by them.

When exploring the role of sex in neonatal outcomes, it is quite obvious that the complexity of the interplay between maternal- placental-offspring factors is closely tied to neonatal sex, especially when focusing on specific neonatal disorders (Madurai et al., Alur et al.). Fetal sex, either secondary to chromosome-specific gene activation/deactivation regulation and/or genetic material, can alter placental functioning, thereby impacting both maternal and fetal health (9, 10). Studies focusing on maternal pregnancy outcomes based on fetal sex have demonstrated a higher cardiovascular and metabolic load for the mother, resulting in poor obstetric outcomes, including but not limited to pregnancy-induced hypertension, gestational diabetes, and preterm birth (11, 12). Male infants have a higher risk of mortality and other morbidities in the neonatal period compared to female infants (13) (Chaudhary and Meharwal). Awareness of these sex-based differential outcomes can help advance the field of personalized medicine, improving prognostication and guiding therapies (14, 15).

While it is well known that race is a social construct, it continues to be reported in medical literature as having biological plausibility, thereby leading to inequities in healthcare provision, utilization, and outcomes (16–19). When delving deeper, it quickly becomes apparent that the differences reported by race are likely secondary to socioeconomic disparities, access to care, and health literacy (Call et al., Konzett et al., Li et al.). By focusing on these modifiable factors through guided resource utilization, an improvement in neonatal outcomes can be achieved (Belay et al., Wogayehu et al.). In high-income countries, there has been a steady improvement in neonatal outcomes that continues to be sustained even with lower limits of viability and more complex neonatal diagnoses (Li et al., Konzett et al.). With the significant advances made in the fields of genetics and epigenetics, it is time to incorporate these biological variables into future research rather than continuing to focus on race-based outcomes (20). Not only will this strategy improve diagnostics, but it will also allow for more targeted therapeutic opportunities (21–23).

Thus, the future of neonatal care improvement warrants not only incorporating personalized medicine methodologies that focus on biological variables when developing therapeutics, but also systematically eradicating the inequities that persist secondary to socio-economic status.
